# Global real‐world evidence of sofosbuvir/velpatasvir as simple, effective HCV treatment: Analysis of 5552 patients from 12 cohorts

**DOI:** 10.1111/liv.14537

**Published:** 2020-06-09

**Authors:** Alessandra Mangia, Scott Milligan, Mandana Khalili, Stefano Fagiuoli, Stephen D. Shafran, Fabrice Carrat, Denis Ouzan, George Papatheodoridis, Alnoor Ramji, Sergio M. Borgia, Heiner Wedemeyer, Ruggero Losappio, Francisco Pérez‐Hernandez, Nicole Wick, Robert S. Brown, Pietro Lampertico, Karen Doucette, Ioanna Ntalla, Heribert Ramroth, Michael Mertens, Kim Vanstraelen, Juan Turnes

**Affiliations:** ^1^ IRCCS Casa Sollievo della Sofferenza San Giovanni Rotondo Italy; ^2^ Trio Health Analytics La Jolla CA USA; ^3^ University of California San Francisco San Francisco CA USA; ^4^ Papa Giovanni XXIII Hospital Bergamo Italy; ^5^ University of Alberta Edmonton Canada; ^6^ Sorbonne Université INSERM Institut Pierre Louis Epidémiologie et Santé Publique APHP.Sorbonne Université Paris France; ^7^ Institut Arnault Tzanck Saint‐Laurent‐du‐Var France; ^8^ National and Kapodistrian University of Athens Athens Greece; ^9^ University of British Columbia Vancouver Canada; ^10^ William Osler Health System Brampton Canada; ^11^ University Clinic Essen Essen Germany; ^12^ Vittorio Emanuele II Hospital Bisceglie Italy; ^13^ Hospital Universitario Nuestra Señora de Candelaria Santa Cruz Spain; ^14^ Weill Cornell Medicine Department of Medicine New York City USA; ^15^ Fondazione IRCCS Ca’ Granda Ospedale Maggiore Policlinico University of Milan Milan Italy; ^16^ Gilead Sciences Europe Ltd Stockley Park United Kingdom; ^17^ Department of Gastroenterology and Hepatology C.H.U. Pontevedra and IIS Galicia Sur Pontevedra Spain

**Keywords:** HCV elimination, Hepatitis C, heterogenous, real‐world, sofosbuvir/velpatasvir, sustained virological response

## Abstract

**Background and aims:**

Achieving sustained virological response (SVR; cure) in hepatitis C patients using a simple regimen is key to making elimination by 2030 possible. In the largest real‐world analysis to date, the effectiveness of pangenotypic, panfibrotic, single‐tablet, sofosbuvir/velpatasvir (SOF/VEL) once‐daily for 12 weeks was assessed in 12 clinical real‐world cohorts from various geographical areas, settings and treatment practices. Factors affecting risk of not achieving SVR were assessed.

**Methods:**

Adults treated with SOF/VEL 400/100 mg, without ribavirin, were included. All HCV patients reaching Week 12 or 24 post‐treatment were assessed for SVR12/24. Factors associated with not achieving SVR12/24 for virological reasons were evaluated using logistic regression analysis.

**Results:**

Overall, 5552 patients were included: 13.3% treatment‐experienced; 20.7% compensated cirrhotic; 30.2% genotype 1; 29.5% genotype 2; 32.9% genotype 3; 4.7% genotype 4; 3.7% HIV coinfection; 13.4% current/former intravenous drug use. Of the 5196 patients evaluated for effectiveness, 98.9% achieved SVR12/24. High SVR12/24 rates occurred in all genotypes including genotype 3 (98.3%; 1649/1677) and in those with compensated cirrhosis (97.9; 1055/1078). Only 55 patients did not achieve SVR12/24 due to a virological reason; the only factor statistically significantly associated with an increased risk of not achieving SVR12/24 was compensated cirrhosis (*P* = .002). Overall, 6% (332/5552) of patients did not achieve SVR12/24 for non‐virological reasons (67% lost to follow‐up; 26.5% early treatment discontinuation).

**Conclusions:**

In this large cohort, representative of clinical practice, a simple 12‐week regimen of SOF/VEL without ribavirin resulted in high SVR12/24 rates in diverse patient populations, even among those with compensated cirrhosis.

**Abbreviations**
AASLD: American Association for the Study of the Liver
DAAs: direct–acting antivirals
EASL: European Association for the Study of the Liver
HCV: hepatitis C virus
HIV: human immunodeficiency virus
LTFU: lost to follow‐up
PPI: proton‐pump inhibitor
SE: standard error
SOF/VEL: sofosbuvir/velpatasvir
SOF/VEL/VOX: sofosbuvir/velpatasvir/voxilaprevir
SVR: sustained virological response
SVR12/24: sustained virological response 12 or 24 weeks after the end of treatment
WHO: World Health Organization

**Key point**

The ability to achieve cure in over 95% of HCV patients treated with direct–acting antiviral agents has made elimination of HCV, a public health priority, a possibility. However, this requires treatment regimens to be simple and well‐tolerated, as well as effective, with very little need for testing before or after treatment. In this large real‐world analysis of data collected globally, once‐daily sofosbuvir/velpatasvir cured almost 99% of patients after a 12‐week course of treatment in a group of patients that reflect the diversity of those infected with HCV in routine clinical practice.

## 1 INTRODUCTION

Hepatitis C virus (HCV) is a major medical and public health concern globally.^1^ The clinical effectiveness, favourable safety profile and high patient tolerability of direct‐acting antivirals (DAAs) have been well reported and cure rates exceed 95% in the DAA treatment era.^2^ Owing to the high cure rates and an associated reduction in liver transplantation, hepatocellular carcinoma and death,^3^, ^4^ DAAs have revolutionized the management of HCV and are recommended by the World Health Organization (WHO) to ensure over 80% of those affected are cured and achieve HCV elimination by 2030.^1^, ^5^ However, despite the availability of these highly effective DAA regimens, approximately 71 million people globally are still waiting to be treated and only 15 countries are currently on track to achieve HCV elimination by 2030.^6^

Recommended DAA regimens can vary in duration, dosing frequency and pill burden, and the requirement for coadministration with ribavirin.^2^, ^7^ The choice of therapeutic regimen can also depend on HCV genotype, previous treatment history and severity of hepatic impairment. Effective pangenotypic regimens that allow the simplification of HCV management, with respect to minimal monitoring, genotyping and assessment of fibrosis stage, have been identified as being essential in ensuring HCV elimination becomes a reality.^8^, ^9^

Sofosbuvir/velpatasvir (SOF/VEL) is the first pangenotypic, panfibrotic, protease inhibitor‐free, all‐oral single‐tablet regimen and can be used as a fixed 12‐week treatment duration in all adult patients with chronic hepatitis C.^10^ The Phase 3 ASTRAL‐1, −2, −3 and −5 trials established the efficacy and safety of SOF/VEL in over 1100 patients with chronic HCV and reported sustained virological response (SVR) rates of 95%‐100% in patients infected with HCV genotypes 1‐6, with or without compensated cirrhosis and irrespective of human immunodeficiency virus (HIV) status, or previous treatment failure with interferon, ribavirin or protease inhibitors.^11^, ^12^, ^13^, ^14^

The expanding use of DAAs in clinical practice in recent years has provided an opportunity to assess their effectiveness and safety in real‐world cohorts, outside the controlled settings of clinical trials. Several real‐world cohorts have evaluated SOF/VEL effectiveness and safety in varying settings, with the results being similar to those of clinical trials.^10^, ^15^, ^16^, ^17^, ^18^, ^19^, ^20^, ^21^, ^22^, ^23^, ^24^

In this integrated real‐world analysis, data from 12 clinical practice cohorts across different real‐world settings in Canada, Europe and the USA were pooled to allow the evaluation of the real‐world effectiveness of SOF/VEL for 12 weeks without ribavirin (based on the label or physician discretion) in the largest available heterogeneous HCV patient population and to investigate any patient characteristics affecting the risk of not achieving SVR.

## 2 PATIENTS AND METHODS

### 2.1 Design and patients

This pooled analysis included data from 12 clinical practice cohorts across Canada, France, Germany, Greece, Italy, Spain and the USA (Table 1). Patients treated with the oral, once‐daily single‐tablet regimen SOF/VEL 400/100 mg for 12 weeks were included. Patients were treated in different clinical settings, including university hospitals, academic centres, community centres, outpatient clinics and private practices. Treatment and patient monitoring were based on local clinical practice and standard of care, at the discretion of the treating physician.

**Table 1:** Overview of cohorts included in analysis

Cohort	Data available	Country	Details of the settings included in each cohort
ANRS‐Hepather	Summary‐level data	France	A multicentre, prospective observational cohort, including patients treated in 32 expert hepatology centres
Borgia	Patient‐level data	Canada	A cohort from a dedicated viral hepatitis clinic in a large community semi‐academic tertiary care hospital equipped with a dedicated nurse and FibroScan^®^
DHC‐R	Summary‐level data	Germany	A cohort from a mixed setting of physicians in private practice (approx. 80%, including Gastro/Hepatology and ID specialists) and University Outpatient Clinics
Greek Cohort	Patient‐level data	Greece	A cohort from outpatient clinics of a tertiary liver centre and one private outpatient clinic
HCV‐TARGET	Patient level data	Europe/USA	A cohort of 44 academic and 17 community centres that provide medical care and antiviral treatment to HCV‐infected patients
HepaC	Patient‐level data	Spain	A multicentre, real‐world prospective observational cohort including patients referred from addition clinics
HELIOS	Patient‐level data	France	A multicentre, real‐world prospective cohort, including patients treated at 46 centres
Mangia	Patient‐level data	Italy	A cohort from private‐research hospitals as well as patients referred from multiple settings, including addiction care centres
NAVIGATORE‐II	Patient‐level data	Italy	A multicentre, real‐world, prospective cohort including patients treated from multiple clinical centres
Ramji	Patient‐level data	Canada	A cohort from an academic tertiary care outside the hospital
Shafran	Patient‐level data	Canada	A cohort from a university hospital with an outpatient building across the street from the hospital. Around 80% of patients were referred by physicians and the rest were self‐referred
TRIO Health	Patient level data	USA	A cohort of healthcare providers and specialty pharmacies

### 2.2 Data collection

Demographics and treatment characteristics were collected at baseline. Reasons for not achieving SVR 12 or 24 weeks after the end of treatment (SVR12/24), for early treatment discontinuation, for being lost to follow‐up (LTFU) and cause of death (if applicable) were evaluated according to local clinical practice standards and documented at the discretion of the treating physician. Patient‐level data were available for 10 cohorts and summary level data for the remaining two cohorts.

### 2.3 Inclusion and exclusion criteria

Adult patients who were treated with SOF/VEL 400/100 mg for 12 weeks with a valid SVR12/24 status or patients who had discontinued treatment early (before the end of February 2019) were included. Inclusion criteria included infection with HCV genotypes 1‐6 and the absence or presence of compensated cirrhosis. Patients who were treatment naïve or had previously received interferon‐based therapy (pegylated interferon plus ribavirin with or without telaprevir, boceprevir or simeprevir) were also included. The following patients were excluded from the analysis: patients who received SOF/VEL for more than 12 weeks or received ribavirin as part of the treatment regimen; patients with current or prior decompensated cirrhosis or hepatocellular carcinoma; and patients who had previously failed a DAA treatment (patients previously treated with pegylated interferon plus ribavirin with or without boceprevir, telaprevir or simeprevir were included).

### 2.4 Outcome assessments

Effectiveness was assessed in the overall population, including all patients with a virological, non‐virological and unknown reason for not achieving SVR12/24. Effectiveness was also assessed in the effectiveness population, which excluded patients who did not have a valid SVR12/24 status because of non‐virological reasons or unknown reasons. Non‐virological reasons were defined as early treatment discontinuation, non‐adherence, reinfection, LTFU, death before SVR assessment, consent withdrawal. Virological reasons were defined as virological breakthrough/non‐response, relapse or virological failure without availability or further details. Patients without information available about a virological or non‐virological reason for not achieving SVR12/24 were classified under ‘unknown reason’. The primary outcome was SVR12/24, which was calculated in the overall and effectiveness populations and stratified by fibrosis stage, previous or current intravenous drug use, and treatment history. Secondly, the effect of patient characteristics on the risk of not achieving SVR12/24 due to virological reasons (relapse, breakthrough, non‐responder) was evaluated.

### 2.5 Statistical analyses

Descriptive characteristics were presented as the number (n) and percentage of patients (%) for the categorical variables. Continuous variables were summarized as mean (standard error; SE). The effect of patient characteristics – compensated cirrhosis (yes vs no), HIV/HCV coinfection (yes vs no), treatment history (treatment naïve vs treatment experienced), HCV genotype (genotype 3 serving as the reference group), proton‐pump inhibitor (PPI) use at baseline (yes vs no), intravenous drug use (yes vs no) – on risk of not achieving SVR12/24 due to virological reasons was evaluated using logistic regression accounting for cohort random effects. Logistic regression analyses were performed in a subset of the effectiveness population with patient‐level data available and no missing data for each characteristic. Significance threshold was set at *P* < .008 (=0.05/6) to account for multiple testing. All analyses were performed using R v3.4.3.

## 3 RESULTS

### 3.1 Patient flowchart and baseline characteristics

**Figure 1:**
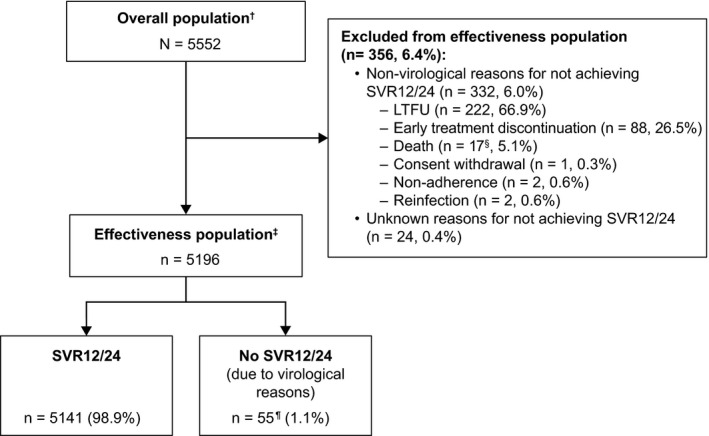
Flowchart and response of patients included in the real‐world effectiveness analysis. ^†^Overall population includes all patients who achieved SVR12/24, and those who did not achieve SVR12/24 due to virological and non‐virological reasons and patients for which the reason for achieving SVR12/24 was unknown. ^‡^Effectiveness population includes all patients who achieved SVR12/24 and those who did not achieve SVR12/24 due to virological reasons. ^§^Two patients died due to sepsis, one due to cancer (not specified further), one due to haemoptysis secondary to primary lung cancer, cause of death was not specified for 13 patients. ^¶^32 patients relapsed, 11 were non‐responders, three were breakthroughs; details of the virological reason were not specified for nine patients, as evaluated and documented by the treating physician. LFTU, lost to follow‐up; SVR12/24, sustained virological response 12/24 weeks after end of treatment

A total of 5552 patients infected with HCV from 12 clinical cohorts (Table 1) who started treatment with SOF/VEL for 12 weeks, without ribavirin, were included in this real‐world pooled analysis (overall patient population). Patients who did not achieve SVR12/24 due to non‐virological (n = 332) or unknown reasons (n = 24) were excluded from the effectiveness population. The patient flowchart including SVR12/24 result is provided in Figure 1. Patient baseline characteristics for the overall patient population and for the effectiveness population are shown in Table 2.

**Table 2:** Baseline demographics and clinical characteristics of HCV‐infected patients for the overall patient population and for the effectiveness population

Characteristics	Overall patient population (N = 5552)	Effectiveness population (n = 5196)
Age		
Mean, years (SE)	56 (2.6)	56 (2.8)
Unknown, n (%)	11 (0.2)	209^a^ (4.0)
Sex, n (%)		
Male	3225 (58.1)	2902 (55.9)
Female	2325 (41.9)	2013 (38.7)
Unknown	2 (0.04)	281^a^ (5.4)
Ethnicity, n (%)		
Caucasian/White	3683 (66.3)	3523 (67.8)
Asian	108 (1.9)	100 (1.9)
Black/African	155 (2.8)	136 (2.6)
Hispanic/Latino	59 (1.1)	52 (1.0)
Other^b^	54 (1.0)	48 (0.9)
Unknown	1493 (26.9)	1337 (25.7)
HIV coinfection, n (%)		
Yes	204 (3.7)	186 (3.6)
No	5101 (91.9)	4766 (91.7)
Unknown	247 (4.4)	244 (4.7)
Intravenous drug use, n (%)		
Former^c^ or current drug use	743 (13.4)	689 (13.3)
No former or current drug use	2058 (37.1)	1992 (38.3)
Unknown	2751 (49.6)	2515 (48.4)
Fibrosis stage^d^, n (%)		
F0‐F2	2984 (53.7)	2839 (54.6)
F3	737 (13.3)	704 (13.6)
F4 (cirrhosis)	1147 (20.7)	1078 (20.7)
No cirrhosis, fibrosis stage unknown	596 (10.7)	493 (9.5)
Fibrosis stage unknown	88 (1.6)	82 (1.6)
PPI^e^, n (%)		
PPI use at baseline	515 (9.3)	478 (9.2)
No PPI use at baseline	3130 (56.4)	2848 (54.8)
Unknown PPI use at baseline	1907 (34.3)	1870 (36.0)
Treatment history, n (%)		
Treatment‐naïve	4815 (86.7)	4521 (87.0)
Treatment history unknown	39 (0.7)	33 (0.6)
Treatment‐experienced (DAA‐naïve)	698 (12.6)	642 (12.4)
PEG‐IFN + ribavirin	484 (69.3)	452 (70.4)
PEG‐IFN + ribavirin +PI	16 (2.3)	15 (2.3)
Multiple previous treatments^f^	3 (0.4)	3 (0.5)
Previous treatment not specified	195 (27.9)	172 (26.8)
HCV genotype, n (%)		
GT 1	1695 (30.5)	1613 (31.0)
GT 1a	495 (29.2)	472 (29.3)
GT 1b	350 (20.6)	329 (20.4)
GT 1 mixed subtype	2 (0.1)	2 (0.1)
GT 1 unknown subtype	848 (50.0)	810 (50.2)
GT 2	1637 (29.5)	1546 (29.8)
GT 3	1825 (32.9)	1677 (32.3)
GT 4	259 (4.7)	239 (4.6)
GT 5‐6	76 (1.4)	68 (1.3)
Mixed GT	11 (0.2)	11 (0.2)
Unknown GT	49 (0.9)	42 (0.8)

Abbreviations: DAA, direct‐acting antiviral; F, fibrosis; GT, genotype; PEG‐IFN, pegylated interferon; PI, protease inhibitor; PPI, proton pump inhibitor; SE, standard error; SOF/VEL, sofosbuvir/velpatasvir.

^a^ For one cohort, only mean age and percentage of male patients was provided, and only for the overall cohort, not for the effectiveness population of that cohort. This results in the number of unknowns being higher in the effectiveness population than in the overall population.

^b^ Other: ethnicity was unspecified (n = 44), Indigenous (n = 4), Hawaiian or other Pacific Islander (n = 3), American‐Indian or Alaska native (n = 2), Egyptian (n = 1), as indicated by the treating physician.

^c^ The definition of former drug use varied between cohorts with respect to timing and this level of detail was not available for most patients.

^d^ Fibrosis score was determined by the treating physician according to local standards of care.

^e^ No information was available on PPI treatment continuation during SOF/VEL treatment, or on dose or type of PPI.

^f^ Patients documented as having received at least one course of PEG‐IFN + ribavirin and one course of PEG‐IFN + ribavirin +PI.

### 3.2 Effectiveness of SOF/VEL for 12 weeks without ribavirin

**Figure 2:**
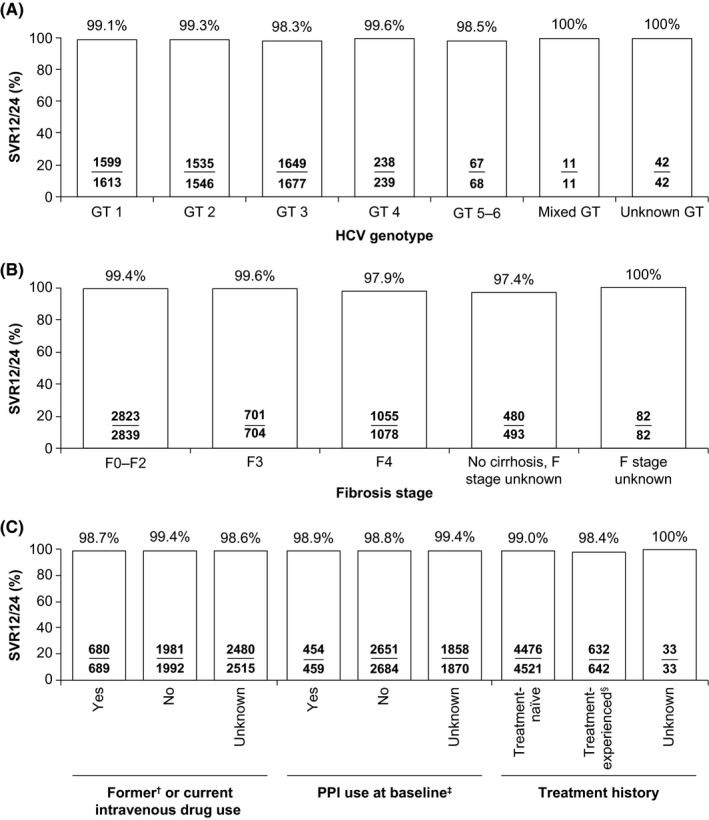
SVR12/24 in the effectiveness population analysis. Percentage of patients achieving SVR12/24 in the effectiveness population after being treated with SOF/VEL for 12 weeks without ribavirin, stratified by (A) HCV genotype (B) fibrosis stage (C) intravenous drug use, PPI use at baseline and treatment history. ^†^The definition of former drug use varied between cohorts with respect to timing and this level of detail was not available for most patients. ^‡^Information on patients achieving SVR12/24 by PPI use at baseline was not available in one cohort, and thus patients from this cohort (n = 183) were not considered in this subgroup analysis. ^§^Patients were treated with PEG‐IFN + RBV (± PI boceprevir, telaprevir, simeprevir). F, fibrosis; GT, genotype; PEG‐IFN, pegylated interferon; PI, protease inhibitor; PPI, proton pump inhibitor; RBV, ribavirin; SOF/VEL, sofosbuvir/velpatasvir; SVR12/24, sustained virological response 12/24 weeks after the end of treatment

In the effectiveness population, SVR12/24 was achieved by 98.9% of patients (5141/5196) (Figure 1), with SVR12/24 being ≥98.3% when stratified by genotype (Figure 2A). Further subgroup analyses demonstrated SVR12/24 rates ≥97.4% when stratified by fibrosis stage (Figure 2B). When stratified by intravenous drug use, treatment history and PPI use SVR12/24 ≥ 98.4% was achieved by all subgroups (Figure 2C).

SVR12/24 rates according to the subtype of patients with HCV genotype 1 were: 98.7% (466/472) for genotype 1a, 98.8% (325/329) for genotype 1b, 100% (2/2) for genotype 1 with mixed subtype and 99.5% (806/810) in genotype 1 with unknown subtype.

SOF/VEL effectiveness data stratified by genotype and presence of compensated cirrhosis was available for 4734 patients from ten cohorts. SVR12/24 rates in these patients with compensated cirrhosis were: 98.3% (349/355) for genotype 1, 98.5% (266/270) for genotype 2, 96.9% (314/324) for genotype 3, 100% (38/38) for genotype 4, 100% (13/13) for genotype 5 and 100% (5/5) for mixed/unknown genotype.

### 3.3 Overall cure rates and reasons for not achieving SVR12/24 with SOF/VEL for 12 weeks

**Figure 3:**
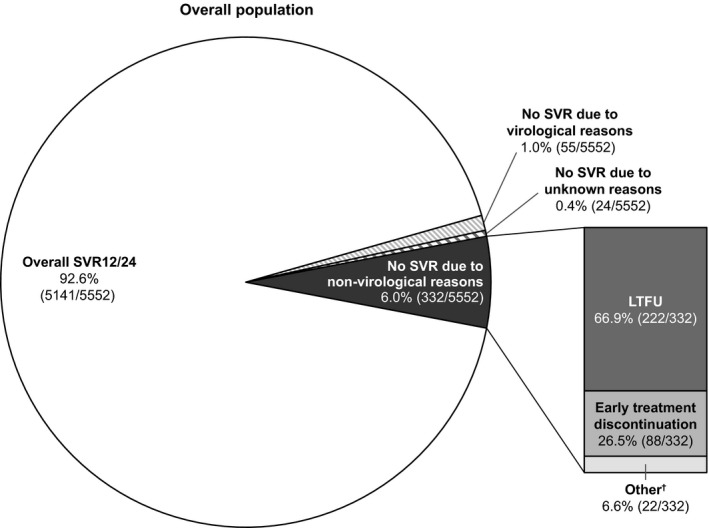
SVR12/24 outcomes in the overall patient population. Treatment outcomes in the overall patient population treated with SOF/VEL for 12 weeks without RBV. ^†^Other non‐virological reasons were: death (5.1%; 17/332), consent withdrawal (0.3%; 1/552), non‐adherence (0.6%; 2/332) and reinfection (0.6%; 2/332). LFTU, lost to follow‐up; RBV, ribavirin; SOF/VEL, sofosbuvir/velpatasvir; SVR12/24, sustained virological response 12/24 weeks after the end of t`reatment

In the overall patient population, SVR12/24 was 92.6% with only 411 patients (7.4%) not achieving SVR12/24:6.0% (332/5552) due to non‐virological reasons and 1.0% (55/5552) due to virological reasons (Figure 3). The reason for not achieving SVR12/24 was unknown in 0.4% (24/5552) of patients. Baseline characteristics of patients who did not achieve SVR12/24 are provided in Table 3.

**Table 3:** Baseline demographic characteristics of patients who did not achieve SVR12/24

Characteristics	Due to virological reasons (n = 55)	Due to non‐virological reasons			Unknown reason (n = 24)
		Total^a^ (n = 332)	LTFU (n = 222)	Early discontinuation (n = 88)	
Age					
Mean, years (SE)	56 (2.1)	52 (2.7)	49 (1.9)	54 (4.3)	51 (10.5)
Sex, n (%)					
Male	35 (63.6)	177 (53.3)	95 (42.8)	46 (52.3)	15 (62.5)
Female	12 (21.8)	102 (30.7)	55 (24.8)	35 (39.8)	9 (37.5)
Unknown	8 (14.5)	53 (16.0)	72 (32.4)	7 (8.0)	0
Ethnicity, n (%)					
Caucasian/White	25 (45.5)	141 (42.5)	87 (39.2)	47 (53.4)	19 (79.2)
Asian	2 (3.6)	8 (2.4)	4 (1.8)	4 (4.5)	0
Black/African	3 (5.5)	17 (5.1)	14 (6.3)	0	2 (8.3)
Hispanic/Latino	1 (1.8)	7 (2.1)	4 (1.8)	2 (2.3)	0
Other^b^	0	6 (1.8)	5 (2.3)	0	0
Unknown	24 (43.6)	153 (46.1)	108 (48.6)	35 (39.8)	3 (12.5)
HIV coinfection, n (%)					
Yes	5 (9.1)	17 (5.1)	8 (3.6)	5 (5.7)	1 (4.2)
No	50 (90.9)	312 (94.0)	211 (95.0)	83 (94.3)	23 (95.8)
Unknown	0	3 (0.9)	3 (1.4)	0	0
Intravenous drug use, n (%)					
Former^b^ or current drug use	9 (16.4)	48 (14.5)	22 (9.9)	7 (8.0)	6 (25.0)
No former or current drug use	11 (20.0)	65 (19.6)	21 (9.5)	7 (8.0)	1 (4.2)
Unknown	35 (63.6)	219 (66.0)	179 (80.6)	74 (84.1)	17 (70.8)
Fibrosis stage^c^, n (%)					
F0‐F2	16 (29.1)	130 (39.2)	52 (23.4)	32 (36.4)	15 (62.5)
F3	3 (5.5)	27 (8.1)	14 (6.3)	8 (9.1)	6 (25.0)
F4 (cirrhosis)	13 (23.6)	103 (31.0)	75 (33.8)	25 (28.4)	0
No cirrhosis, fibrosis stage unknown	23 (41.8)	66 (19.9)	29 (13.1)	22 (25.0)	3 (12.5)
Fibrosis stage unknown	0	6 (1.8)	52 (23.4)	1 (1.1)	0
PPI^d^, n (%)					
PPI use at baseline	5 (9.1)	59 (17.8)	15 (6.8)	14 (15.9)	0
No PPI use at baseline	33 (60.0)	249 (75.0)	115 (51.8)	67 (76.1)	11 (45.8)
Unknown PPI use at baseline	17 (30.9)	24 (7.2)	92 (41.4)	7 (8.0)	13 (54.2)
Treatment history, n (%)					
Treatment‐naïve	45 (81.8)	274 (82.5)	141 (63.5)	81 (92.0)	20 (83.3)
Treatment history unknown	0	6 (1.8)	56 (25.2)	1 (1.1)	0
Treatment‐experienced (DAA‐naïve)^e^	10 (18.2)	52 (15.7)	25 (11.3)	6 (6.8)	4 (16.7)
HCV genotype, n (%)					
GT 1	14 (25.5)	76 (22.9)	27 (12.2)	27 (30.7)	6 (25.0)
GT 2	11 (20.0)	84 (25.3)	48 (21.6)	23 (26.1)	7 (29.2)
GT 3	28 (50.9)	137 (41.3)	85 (38.3)	32 (36.4)	11 (45.8)
GT 4	1 (1.8)	20 (6.0)	5 (2.3)	2 (2.3)	0
GT 5‐6	1 (1.8)	8 (2.4)	2 (0.9)	2 (2.3)	0
Mixed genotype	0	0	0	0	0
Genotype unknown	0	7 (2.1)	55 (24.8)	2 (2.3)	0

Abbreviations: DAA, direct‐acting antivirals; GT, genotype; PEG‐IFN, pegylated interferon; PPI, proton pump inhibitor; RBV, ribavirin; SOF/VEL, sofosbuvir/velpatasvir; SVR12/24, sustained virological response 12/24 weeks after the end of treatment.

^a^ Other non‐virological failures included death (n = 17, 4.8%), consent withdrawal (n = 1, 0.3%), non‐adherence (n = 2, 0.6%) and reinfection (n = 2, 0.6%).

^b^ The definition of former drug use varied between cohorts with respect to timing and this level of detail was not available for most patients.

^c^ Fibrosis score determined by the treating physician.

^d^ No information available on PPI treatment continuation or discontinuation during SOF/VEL treatment.

^e^ Patients were treated with PEG‐IFN + RBV (± PI boceprevir, telaprevir, simeprevir).

### 3.4 Patients who did not achieve SVR12/24 due to a non‐virological reason

The main non‐virological reasons for not achieving SVR12/24 were being LTFU (66.9%; 222/332) and early treatment discontinuation (26.5%; 88/332). Of the 222 patients who were LTFU, timing of when this occurred was available for 67.6% patients (150/222): 40% (60/150) were LTFU during treatment and 60% (90/150) between end of treatment and post‐treatment week 12 or 24.

The reasons for discontinuing treatment early were available in 17% (15/88) of patients: three discontinued treatment because they were denied insurance or could not afford treatment; one moved to another country; three were non‐adherent; and eight discontinued because of adverse events (not otherwise specified) or fear of adverse events.

### 3.5 Patients who did not achieve SVR12/24 due to virological reasons

In the overall patient population, 1.0% (55/5552) of patients did not achieve an SVR12/24 due to a virological reason (Figure 3). Retreatment information was available for 23 patients. Of these, 83% (19/23) patients were retreated; 89% (17/19) successfully. Of the four patients who were not retreated, three were diagnosed with hepatocellular carcinoma and one died before retreatment could be started (cause of death was not provided). The following regimens were used: sofosbuvir/velpatasvir/voxilaprevir (SOF/VEL/VOX; 17/19), SOF/VEL/VOX plus ribavirin (1/19) and glecaprevir/pibrentasvir plus sofosbuvir (1/19).

Logistic regression analyses were performed in a pooled subset of patients from the effectiveness population with individual level data available, to investigate the effect of patient characteristics on not achieving SVR12/24 due to virological reasons. Genotype, intravenous drug use, PPI use at baseline, treatment history and HIV/HCV coinfection were not significantly associated with the risk of not achieving SVR12/24 after controlling for cohort random effects (*P* > .05; Table 4). Only compensated cirrhosis was associated with an increased risk of not achieving SVR12/24 due to virological reasons (OR = 2.53, 95% CI 1.38‐4.55, *P* = .002; Table 4). Among patients with compensated cirrhosis included in this analysis, 2% (20/985) did not achieve SVR12/24; 0.7% (27/3640) of patients without compensated cirrhosis did not achieve SVR12/24.

**Table 4:** Effect of patient characteristics on the risk of not achieving SVR12/24 due to virological reasons

Characteristics	No SVR/SVR12/24 (%)	Odds ratio	95% CI	*P*
HCV genotype				
GT 3	22/1518 (1.4)	Ref.	—	—
GT 1	12/1444 (0.8)	0.84	0.39‐1.70	.634
GT 2	11/1434 (0.8)	0.66	0.30‐1.38	.285
GT 4	1/193 (0.5)	0.61	0.03‐3.01	.629
GT 5‐6	1/50 (2.0)	0.86	0.05‐4.41	.881
Compensated cirrhosis				
No	27/3640 (0.7)	Ref.	—	—
Yes	**20/985 (2.0)**	**2.53**	**1.38**‐**4.55**	**.002**
Intravenous drug use^a^				
No	8/1666 (0.5)	Ref.	—	—
Yes	4/541 (0.7)	1.35	0.34‐4.49	.642
PPI use at baseline				
No	30/2389 (1.3)	Ref.	—	—
Yes	5/445 (1.1)	1.08	0.36‐2.62	.870
Treatment history				
Treatment‐naïve	39/4124 (0.9)	Ref.	—	—
Treatment‐experienced	8/530 (1.5)	1.28	0.54‐2.66	.543
HIV/HCV coinfection				
No	43/4267 (1.0)	Ref.	—	—
Yes	4/176 (2.3)	2.71	0.78‐7.26	.072

Note

Abbreviations: 95% CI, 95% confidence interval; GT, genotype; PPI, proton pump inhibitor; Ref., reference group; SVR12/24, sustained virological response 12/24 weeks after end of treatment.

Logistic regression analyses adjusted for cohort random effects were performed in a pooled subset of the effectiveness population with individual‐level data available and no missing data for each characteristic.

^a^ Current or past intravenous drug use. The definition of former drug use varied between cohorts with respect to timing and this level of detail was not available for most patients.

## 4 DISCUSSION

In this pooled analysis of real‐world clinical practice cohorts, over 5000 patients with HCV infection completed treatment with SOF/VEL for 12 weeks and achieved an SVR12/24 rate of 98.9%, which is in line with previous clinical trial results.^11^, ^12^ This is the largest real‐world cohort of patients treated with DAAs to date, which allowed effectiveness to be analysed in multiple patient subgroups. SVR12/24 rates were high despite inclusion of diverse patient types from different geographical regions, treated in different clinical settings with a variety of management protocols for pre‐ and on‐treatment monitoring and testing. The low discontinuation (<2%) and LTFU rates (4%) in this real‐world analysis are consistent with previous clinical studies.^11^, ^12^ Additionally, where information was available, few of the discontinuations were due to adverse events linked to SOF/VEL therapy, which is consistent with the Phase 3 data. This underlines the favourable safety and tolerability profile of SOF/VEL as a protease inhibitor‐free DAA, as also shown previously in clinical trials. The virological failure rates was very low at 1.0% (55/5552) and similar to the rate of 1.2% (20/1690) observed in the combined ASTRAL‐1,2,3,5 and POLARIS‐2,3 Phase 3 clinical trials.^11^, ^12^, ^14^, ^25^

SVR12/24 rates in the full analysis of 12 cohorts were all 96.9% or higher and similar to previously reported cure rates across multiple patient populations, including treatment‐naïve patients, patients with compensated cirrhosis, and patients with current or former drug use.^11^, ^12^, ^26^, ^27^, ^28^ High SVR12/24 rates were achieved across all genotypes, including genotype 3, which was not associated with a higher risk of not achieving SVR12/24 due to virological reasons. Of note, this cohort includes the largest number of patients infected with genotype 3 reported so far in a real‐world data analysis. Of the 1677 patients with genotype 3 infection, 98.3% were cured without ribavirin. These results are in line with the Phase 3 ASTRAL‐3 and POLARIS‐3 trials which reported SVR12 rates between 93% and 98% in non‐cirrhotic and cirrhotic patients.^12^, ^25^

As the focus of HCV management moves towards elimination, the need for simplified treatment and patient management – with a reduced need for pretreatment and on‐treatment testing – is considered a key step in achieving the WHO goals, by allowing a shorter time between HCV diagnosis and treatment start, thus encouraging a test‐and‐treat approach.^8^, ^29^, ^30^ This is also endorsed in the call to action to work towards HCV elimination launched by the American Association for the Study of Liver Diseases (AASLD)/European Association for the Study of the Liver (EASL)/Asian Pacific Association for the Study of the Liver/Latin American Association for the Study of the Liver and presented during the AASLD Liver Meeting in November 2019.^31^ All patients with unknown genotype, unknown fibrosis score and unknown treatment history in this large patient cohort were cured with SOF/VEL for 12 weeks, offering the possibility of using this regimen in simplified management protocols without the need for extensive pre‐ or on‐treatment monitoring.

Achieving high cure rates in this real‐world setting, without the addition of ribavirin, confirms that ribavirin may not be necessary to cure HCV in populations where physicians may previously have considered its use, including patients with genotype 3 and with compensated cirrhosis.^2^, ^32^, ^33^ Use of a ribavirin‐free regimen also supports the simplification of treatment by reducing the need for monitoring of ribavirin‐related adverse events.

The large size of this real‐world cohort allowed evaluation of one of the few remaining questions in clinical management of HCV patients: are there any factors that predict the risk of not achieving an SVR? Of the 5552 patients treated with SOF/VEL, only 1% did not achieve SVR12/24 due to known virological reasons. In this small subset of patients, only the presence of compensated cirrhosis was significantly associated with not reaching SVR12/24 due to a virological reason. Other factors, including genotype 3, were not significantly associated with not reaching SVR12/24. Small numerical differences are often not statistically significant in smaller studies. In contrast, in this real‐world analysis a difference of 1.3% was statistically significant because of the size of the cohort. However, as high response rates were achieved both in patients without cirrhosis (99.2%) and with compensated cirrhosis (97.9%), this statistically significant difference is unlikely to have a clinical impact, especially when considering that a large majority of people with HCV infection do not have cirrhosis. Retreatment data were available in a subset of 19 patients who had not achieved SVR12/24 with SOF/VEL due to virological reasons; 89% responded to subsequent therapy. Most patients received SOF/VEL/VOX for 12 weeks, which is line with the current treatment recommendations.^2^, ^5^, ^7^

In addition to the low number of patients that did not achieve SVR12/24 due to virological reasons, only 6.7% of patients did not achieve SVR12/24 due to a non‐virological reason, which is consistent with previously reported data in the current DAA era.^34^, ^35^ The majority of these patients were LTFU (67%, 222/332). It is likely that many of these patients were cured, but there is no way of confirming this. This result encourages the possibility of implementing broad HCV elimination strategies; however, it also highlights that there is still room for further improvement of all stages of the care cascade. Simplifying and decentralizing HCV care, by task sharing and integration of HCV care outside the specialist setting,^31^ has been proven to be effective at increasing screening and linkage to care and improving the care cascade.^34^

Despite the large size of this real‐world patient cohort, a few limitations could be identified. Firstly, there is an inevitable amount of missing data because of differences in clinical practice regulations and protocols across the cohorts, in part due to the retrospective data collection. However, high SVR12/24 rates were demonstrated in all patient subsets, even in those with missing values for characteristics such as cirrhosis status, genotype subtype, treatment history and intravenous drug use, minimising the impact of these missing data. Although consistent details were not available for all patients, such as fibrosis assessment methodology, reliable clinical expertise enabled effective staging of patients. It is also important to acknowledge the lack of virological outcome data in patients LTFU or discontinued early, but this is similar to the situation seen in clinical studies. Secondly, another limitation of this study was the inability to assess the SVR in the cohorts with decompensated cirrhosis as the standard‐of‐care recommendations suggest use of ribavirin, which was not the goal of this study. In addition, not all cohorts combined uniformly collected evaluable data in these settings. Nevertheless, we were able to show near universal response rates in those with advanced but compensated liver disease. Furthermore, as only patients treated with SOF/VEL for 12 weeks without ribavirin were included, patient selection bias could be considered a limitation of this analysis. However, the patient demographics show a diverse patient population, with the inclusion of patients traditionally considered ‘difficult‐to‐treat’ or ‘difficult‐to‐cure’, demonstrating a limited selection bias.

To summarize, this large, multicentre, real‐world, diverse, clinical practice cohort emphasizes the strength of SOF/VEL as a simple and effective pangenotypic, panfibrotic DAA regimen, that requires only minimal monitoring and could be applicable in a test‐and‐treat approach to progress towards the goal of HCV elimination.

## CONFLICT OF INTEREST

Alessandra Mangia: Research grants from Gilead Sciences, Merck Sharp and Dohme, Janssen and Bristol‐Myers Squibb. Part of the speaker bureau for Gilead Sciences, Intercept and Merck Sharp and Dohme. Scott Milligan: Research support from Gilead Sciences, during the conduct of the study. Research support from Gilead Sciences, AbbVie and Merck Sharp and Dohme, outside the submitted work. Mandana Khalili: Grants from HCV‐TARGET study sponsors, during the conduct of the study. HCV‐TARGET is an investigator‐initiated study jointly sponsored by The University of Florida, Gainesville, FL (PI: Nelson), and The University of North Carolina at Chapel Hill, Chapel Hill, NC (PI: Fried) and is funded in part by AbbVie, Bristol Myers Squibb, Gilead, GlaxoSmithKline, Janssen, Kadmon, and Merck. Grants from Gilead Sciences, Intercept Pharmaceuticals and AbbVie, outside of submitted work. Personal fees from Gilead Sciences, outside the submitted work. Stefano Fagiuoli: Grants and personal fees from Gilead Sciences, AbbVie and Novartis, outside the submitted work. Personal fees from Merck Sharp and Dohme, Astellas and Bayer, outside the submitted work. Stephen Shafran: Grants and personal fees from Gilead Sciences, during the conduct of the study. Grants from AbbVie, Merck Sharp and Dohme, and Janssen, outside the submitted work. Fabrice Carrat; Grants from Inserm‐ANRS, during the conduct of the study. Denis Ouzan: Grants from Gilead Sciences, during the conduct of the study. Grants, personal fees and non‐financial support from Gilead Sciences and AbbVie, outside the submitted work. George Papatheodoridis: Grants from Gilead Sciences, during the conduct of the study. Grants from Gilead Sciences and AbbVie, outside of submitted work. Personal fees and non‐financial support from Gilead Sciences, AbbVie and Merck Sharp and Dohme, outside of submitted work. Alnoor Ramji: Grants from AbbVie, Gilead Sciences, Janssen, Novartis, Merck and Springbanks, outside the submitted work. Other from AbbVie, Allergen, Assembly, Celgene, Gilead Sciences and Janssen. Sergio Borgia: Nothing to disclose. Heiner Wedemeyer: Grants and personal fees from Gilead Sciences, during the conduct of the study. Grants, personal fees and non‐financial support from Abbott and Roche Diagnostics, outside the submitted work. Grants and personal fees from Bristol Myers Squibb, Novartis, Roche Diagnostics, Merck Sharp and Dohme, Eiger and Falk and Falk Foundation, outside the submitted work. Personal fees from Siemens and Janssen, outside the submitted work. Investigator for Transgene, outside the submitted work. Non‐financial support from MYR‐GmbH, outside the submitted work. Ruggero Losappio: Nothing to disclose. Francisco Pérez‐Hernandez: Personal fees from Gilead Sciences and AbbVie, outside the submitted work. Nicole Wick: Research support from Gilead Sciences, during the conduct of the study. Research support from AbbVie and Merck, outside the submitted work. Robert S Brown Jr: Grants and personal fees from Gilead Sciences, during the conduct of the study. Grants and personal fees from AbbVie, outside of submitted work. Pietro Lampertico: Personal fees from Gilead Sciences, AbbVie and Merck Sharp and Dohme, outside the submitted work. Karen Doucette: Clinical trials and honoraria from Gilead Sciences and unrestricted educational grant from Merck, outside the submitted work. Ioanna Ntalla, Heribert Ramroth, Michael Mertens and Kim Vanstraelen: Employees and stock owners of Gilead Sciences, during the conduct of the study and outside of submitted work. Juan Turnes: Personal fees from AbbVie, outside the submitted work. Personal fees and grant from Gilead Sciences, outside the submitted work.
